# Fracture resistance of endocrowns made from different CAD/CAM materials after prolonged fatigue aging

**DOI:** 10.1007/s00784-025-06241-4

**Published:** 2025-02-22

**Authors:** Arslandaş Dinçtürk B, Sufyan Garoushi, Kedici Alp C, Vallittu PK, Üçtaşlı MB, L Lassila

**Affiliations:** 1 Department of Restorative Dentistry, Faculty of Dentistry, University of Gazi, Ankara, Turkey; 2 Department of Biomaterials Science and Turku Clinical Biomaterial Center - TCBC Institute of Dentistry, University of Turku, Turku, Finland; 3 Wellbeing Services County of South-West Finland, Turku, Finland

**Keywords:** Endocrown restoration, CAD/CAM, Fracture resistance, Fiber reinforced composite

## Abstract

**Objectives:**

The aim of this study was to evaluate the fracture resistance of endocrowns made of experimental short fiber-reinforced CAD/CAM block (SFRC CAD) in comparison with different commercial CAD/CAM materials.

**Materials and methods:**

In this study, 60 sound mandibular molar teeth with similar occlusal sizes (± 1 mm) were used. Standard preparations simulating an endocrown cavity were prepared and the teeth were divided into four groups randomly. After the digital photoimpression of the prepared cavities using a dental intraoral scanner (CEREC), the restorations were designed (4 mm high) and milled using IPS e.max, Cerasmart 270, Katana Avencia and SFRC CAD blocks (*n* = 15/per group). Restorations were cemented with self-adhesive dual-cure resin cement (G-Cem One). The specimens were immersed in a 37 °C water bath within the chewing simulator. Following cyclic fatigue aging for 1.000.000 cycles (F_max_=150 N), a quasi-static load was applied using a universal testing machine at a speed of 1 mm/min. Fractography analysis was conducted using optical microscopy.

**Results:**

According to the data obtained, there are statistically significant differences in fracture resistance values between different CAD/CAM materials (*p* < 0.05). The highest values were observed in the experimental SFRC CAD group (3025 N), while the lowest values were observed in the IPS e.max group (2295 N).

**Conclusions:**

The type of CAD/CAM restorative material influences the fracture resistance of endocrowns. SFRC CAD blocks could potentially serve as an alternative material for endocrown restorations in the future.

**Clinical relevance:**

Endocrowns fabricated from SFRC CAD blocks have demonstrated promising fracture behavior, suggesting their suitability for clinical testing.

## Introduction

Mechanical and biological changes occur in teeth undergoing endodontic treatment due to caries, endodontic access cavities, trauma, previous restorations, and processes of root canal preparation. One of the significant variables that directly impacts the prognosis of endodontic treatment is the coronal restoration of these teeth. When teeth with one or more lost walls undergo endodontic treatment, the tooth becomes less resistant to occlusal forces, which increases the risk of crown damage [1].

To prevent endodontically treated teeth (ETT) fractures, it is crucial to strengthen the teeth intracoronally, particularly in the posterior region [2, 3]. For ETT with significant tissue loss, post and cores, ceramic inlays and onlays, and endocrown restorations were suggested as treatment options [4–6]. Studies have shown that endocrowns are the most preferred treatment modality for restoring endodontically treated teeth (ETT) in patients with occlusal considerations [7]. As monolithic endocrowns have superior fracture resistance when compared to those restored with hybrid post/core/crown [8]. Over a 3–19 years follow-up, recent systematic reviews and meta-analyses demonstrate high success rates of 72–99% for endocrowns in molars [9]. According to Al-Dabbagh [7], endocrowns have demonstrated survival and success rates comparable to those of conventional post-retained crown restorations, suggesting that they are a reliable choice for treating molars.

Utilizing chairside CAD/CAM processing expedites manufacturing and eliminates the need for a temporary restoration. Additionally, it allows for high-quality patient rehabilitation in a single visit [10]. The success of such restoration depends on the properties of the materials used, the restoration technique, clinician’s skill, and the surrounding tissues’ health [10, 11]. Ideal characteristics of the restorative material for ETT are its resistance to occlusal forces, ability to prolong the survival of the restored tooth and ability to generate visually appealing outcomes [12, 13]. Different CAD/CAM materials like full ceramic materials, feldspathic ceramics, lithium disilicate, leucite glass ceramics, zirconium reinforced glass ceramics, resin nanoceramics and polymer infiltrated ceramic blocks can be used for ETT restoration [14].

CAD/CAM hybrid ceramics and resin composites have a lower elastic modulus closer to that of dentin. Under clinical loads, these hybrid materials can function as stress absorbers and reduce stress peaks at the interfaces between restorations and teeth [15, 16]. Comparing CAD/CAM resin composites to lithium disilicate ceramics, a recent systematic review of in vitro research revealed that the CAD/CAM resin composites had greater fracture resistance and damage tolerance and fewer catastrophic failures [17]. However, the efficiency of CAD/CAM resin composites remains controversial, as their mechanical properties can be inferior to those of ceramics [18].

Clinically, fractures in resin composite-based endocrowns have been observed due to crack propagation, leading to the breakdown of the entire composite structure. These fractures mostly started in the central occlusal groove, indicating that these restorations have lower toughness and fatigue resistance [12, 13, 19].

In order to reinforce the mechanical characteristics of conventional CAD/CAM resin composites and mitigate potential problems that could negatively impact their long-term clinical performance, experimental short fiber-reinforced CAD/CAM block (SFRC CAD) has been developed [20, 21]. The goal of introducing SFRC CAD composite blocks was to enhance the fracture toughness of conventional particulate-filled CAD/CAM composites. Research on their optical, mechanical, bonding, and surface properties has shown promising results [22–25]. However, there is no data regarding the loading performance of SFRC CAD when used to fabricate endocrowns. Therefore, the purpose of this study is to evaluate the effect of experimental SFRC CAD on the fracture behaviour of endocrown restorations compared to commercial CAD/CAM materials following prolonged fatigue aging. The study’s null hypothesis is that SFRC CAD endocrown restorations’ loading behavior will be the same as that of other CAD/CAM endocrown restorations.

## Materials and methods

The materials used in this study, along with their composition, are listed in Table 1.

**Table 1:** CAD/CAM materials used in the study

Material	Manufacturer	Composition according to manufacturer
IPS e.max	Ivoclar Vivadent AG, Schaan Liechtenstein	Silicon dioxide 57–80%, Lithium oxide 11–19%, Potassium oxide 0–13%, Phosphorus oxide 0–11% and other oxides
Cerasmart 270	GC Corp, Tokyo, Japan	Bis-MEPP, UDMA, dimethacrylate, Silica (20 nm), barium glass (300 nm) 71 wt%
Katana Avencia	Kuraray Noritake Dental, Japan	UDMA, dimethacrylate, colloidal silica and aluminum oxide 62 wt%
SFRC CAD	Experimental	UDMA, TEGDMA, Short glassfiber (200–300 μm & Ø7 µm), Barium glass 77 wt%

TEGDMA, triethylene glycol dimethacrylate; UDMA, urethane dimethacrylate; Bis-MEPP, Bis (p-methacryloxy (ethoxy) 1–2 phenyl)-propane; wt%, weight percentage

The composition of the experimental SFRC CAD block was as follows: the monomer mix, comprising 23 wt%, consisted of 70% UDMA and 30% TEGDMA. Short fibers accounted for 25 wt%, while barium glass fillers made up 52 wt%. The fabrication process involved mixing the monomers with photoinitiators, fillers, and fibers to create a homogeneous blend (Thinky Mixer ARV-310, TM, Tokyo, Japan). This mixture was then placed into rectangular molds and compacted using a pressure kettle to enhance density and eliminate voids. Polymerization was performed under controlled conditions, utilizing heat up to 170 °C and high pressure to ensure optimal monomer conversion. Once cooled, the blocks were trimmed to precise dimensions to fit the CEREC machine (Sirona Dental Systems Inc., Long Island City, NY), and holding pins were bonded to the lower surface of the block for compatibility during milling.

**Fig. 1:**
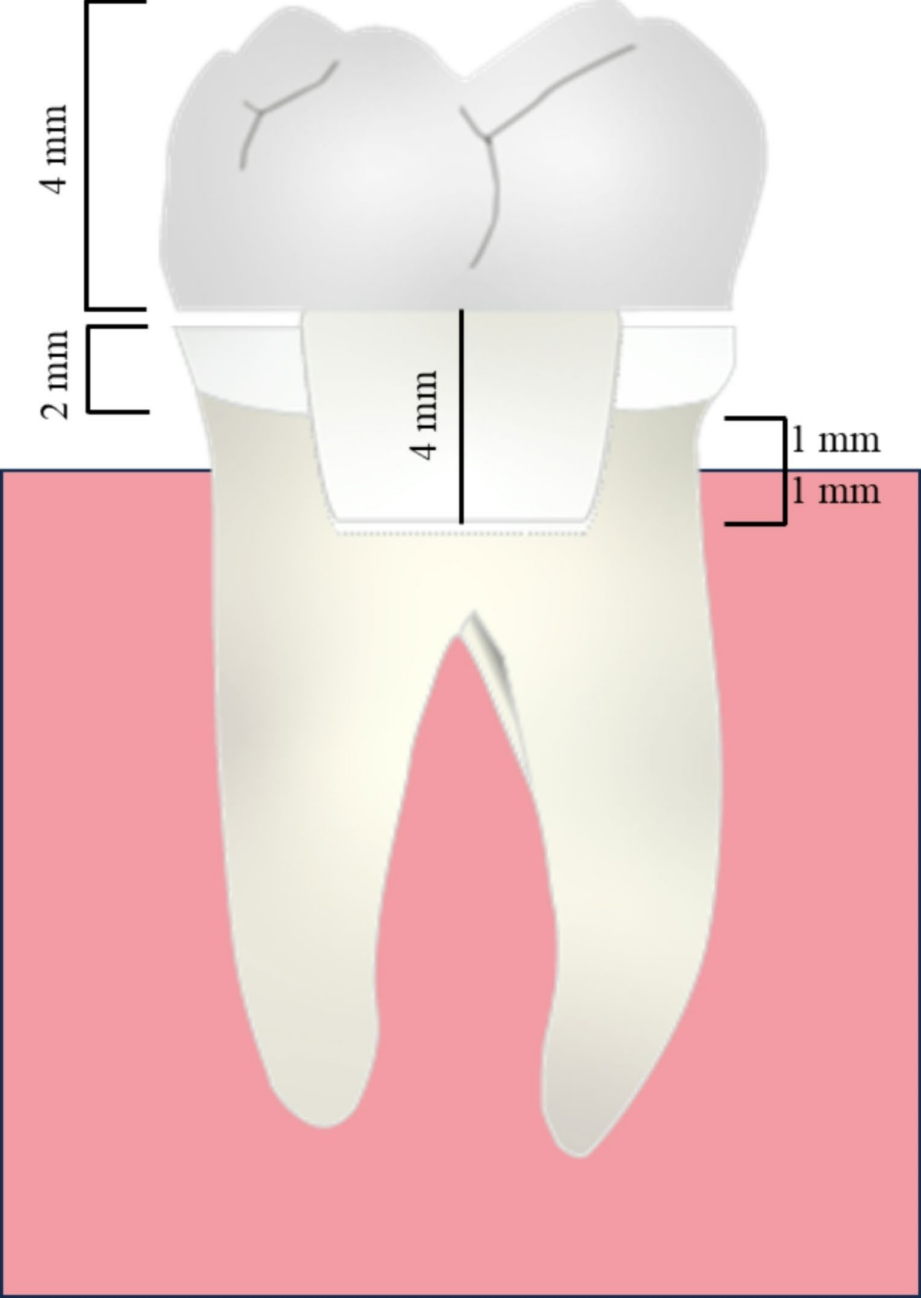
Schematic diagram illustrating tooth preparation and endocrown measurements in millimeters

60 sound mandibular molar teeth with occlusal sizes averaging ± 1 mm were utilized. Teeth were collected with permission obtained from the Institute of Dentistry, University of Turku, Finland, in accordance with the law No. 101/2001, Sect. 20, Act on the Medical Use of Human Organs, Tissues and Cells. The selected teeth were kept in 0.5% chloramine T solution for a one month following the elimination of soft tissues under water. Digital caliper (Mitutoyo Corp., Tokyo, Japan) was used to measure every tooth’s size from various angles. In the mesio-distal and bucco-lingual directions, mean diameters were 11.3 (± 0.6) and 10.3 (± 0.5) respectively. After that, auto-polymerized acrylic resin was used to embed the teeth from 1 mm beneath the cement-enamel junction on an acrylic block (diameter of 2.5 cm). Every tooth received a coronal preparation that was comparable (Fig. 1). There was only a single operator who performed the tooth preparation and restorations.

### Teeth preparation and restorative techniques

Using a high-speed handpiece under water cooling, round-end diamond burs (850–014 M SSWhite, Lakewood, NJ, USA) and flat-end parallel carbide (H21LR.314.010, Brasseler, Savan- nah, GA, USA), standard preparations simulating an endocrown cavity were created. The preparation was carried out from the occlusal surface to obtain a flat cavity floor and preparation was made 2 mm beyond the cement-enamel junction. For pulp chamber preparations, 4*3 mm^2^ cavities were made. Following cavity preparation, the tooth surfaces were prepared with 37% phosphoric acid and a universal bonding agent (G2-Premio Bond, GC Corp., Japan) in accordance with the manufacturer’s instructions. After that, teeth were divided into four groups randomly.

After obtaining a digital photoimpression of the prepared cavity using a dental intraoral scanner (CEREC), the restorations were designed with a standardized height of 4 mm and milled from IPS e.max, Cerasmart 270, Katana Avencia, and SFRC CAD blocks (*n* = 15). The occlusal design adhered to a standardized digital workflow, replicating the morphology of a mandibular first molar to ensure consistent contact points. Prior to cementation, as per the manufacturer’s instructions, the inner surfaces of all restorations except for those in the IPS e.max group were etched for 60 s using IPS Ceramic Etching Gel (Ivoclar Vivadent AG, Liechtenstein) containing 4.5% hydrofluoric acid. IPS e.max restorations were etched for 20 s.

The next following steps involved washing and air-drying. Self-adhesive dual cure resin cement (G-Cem One, GC, Japan) and Multi primer (G-Multi Primer, GC, Japan) were used for luting the restorations. With hand-light curing unit (Elipar TM S10, 3 M ESPE, Germany) to light-cure each segment for 20 s in all directions. The endocrown restorations were very close to the source of light. Before being tested, restorations submerged in water at 37 °C for two days.

### Cyclic fatigue aging

Before the quasi-static fracture load test, the restored teeth underwent cyclic fatigue aging. Using a chewing simulator (MOD, Esetron Smart Robotechnologies, Ankara, Turkey), the specimens were immersed in a 37 °C water bath and subjected to 1.000.000 cycles of mechanical loading with a metal ball (Ø 6 mm) at a frequency of 1.5 Hz and a maximum force of 150 N. The loading ball was in contact with the triangular ridges of the lingual and buccal cusps. The specimens remained in water for a total of eight weeks, both during and after cyclic aging.

### Fracture load test

**Fig. 2:**
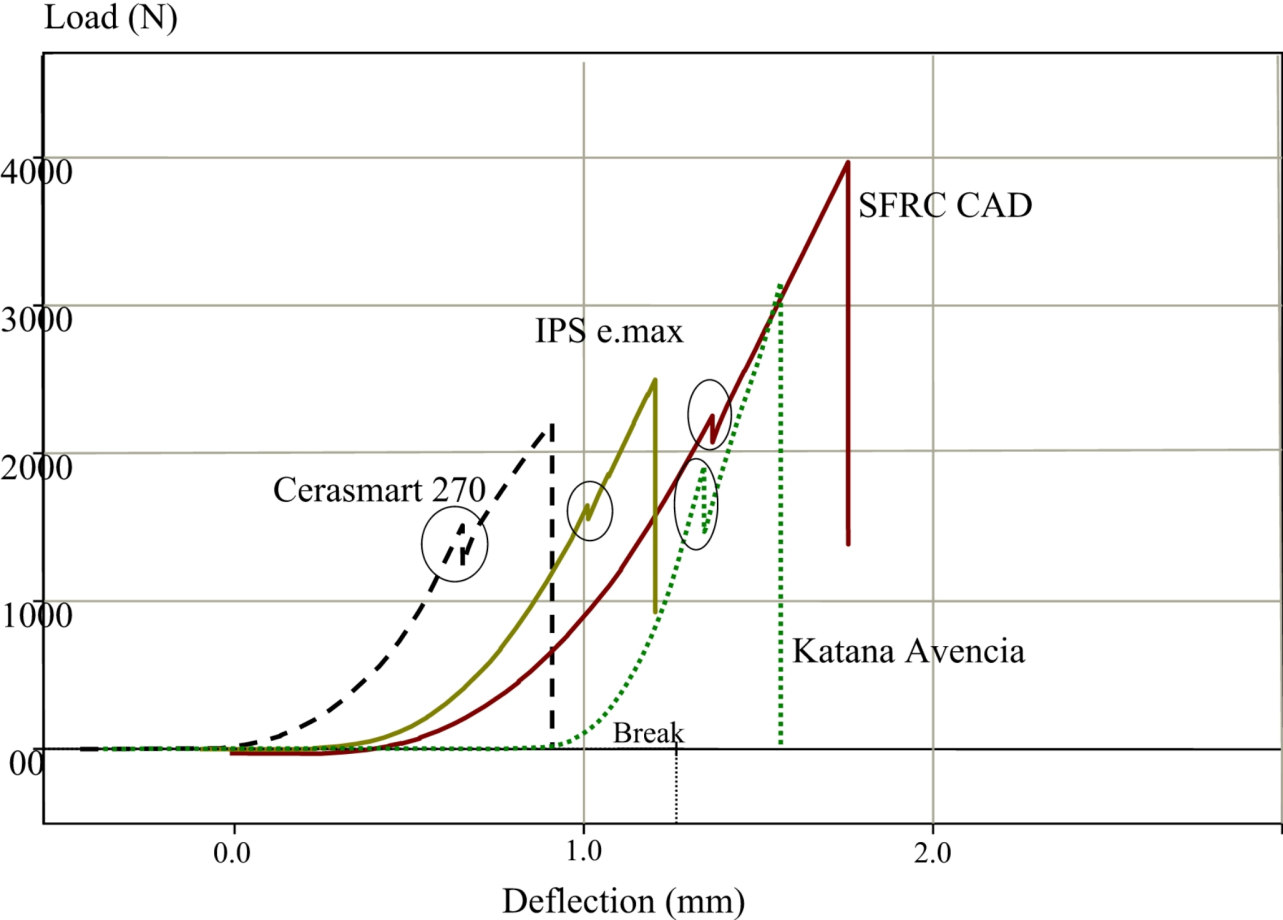
Load–deflection curve of representative specimens from endocrown restorations showing the first drop in the curves (circle peaks) which considered as initial failures before the final failure drop

Using a universal testing machine (Lloyd model LRX, UK) at a speed of 1 mm/min, a quasi-static load was applied after cyclic fatigue aging. A metal ball with a diameter of 5 mm was used to deliver the load vertically, making contact with the triangular ridges of the lingual and buccal cusps, creating three points of contact. Restorations were subjected to the loading force until they fractured. The initial and final fracture load values were derived from load–displacement curve. The maximum or final fracture load was established through visual examination of structural damage in conjunction with the load–displacement curve (Fig. 2). The area under the load-displacement curve of fractured specimens was used to calculate the work of fracture and the result was reported in Ncm.

### Fracture mode analysis

The fracture mode of the restorations was assessed through visual inspection and stereomicroscopy at different magnifications and illumination angles (Heerbrugg M3Z, Heerbrugg, Switzerland). After independently examining the specimens, three researchers reached a consensus on the type, location, and direction of failure. Based on the visual inspection of each loaded restoration’s fracture mode, the fractures were categorized into two groups: catastrophic fractures, which damaged both the restoration and the tooth structure, and fractures involving only the restoration (repairable).

### Statistical analysis

Using SPSS version 13 (Statistical Package for Social Science, SPSS Inc, Chicago, IL, USA), data were statistically assessed using analysis of variance (ANOVA) (*P* < 0.05). Additional multiple comparisons were performed using Tukey’s post hoc method and pairwise comparison.

## Results

**Fig. 3:**
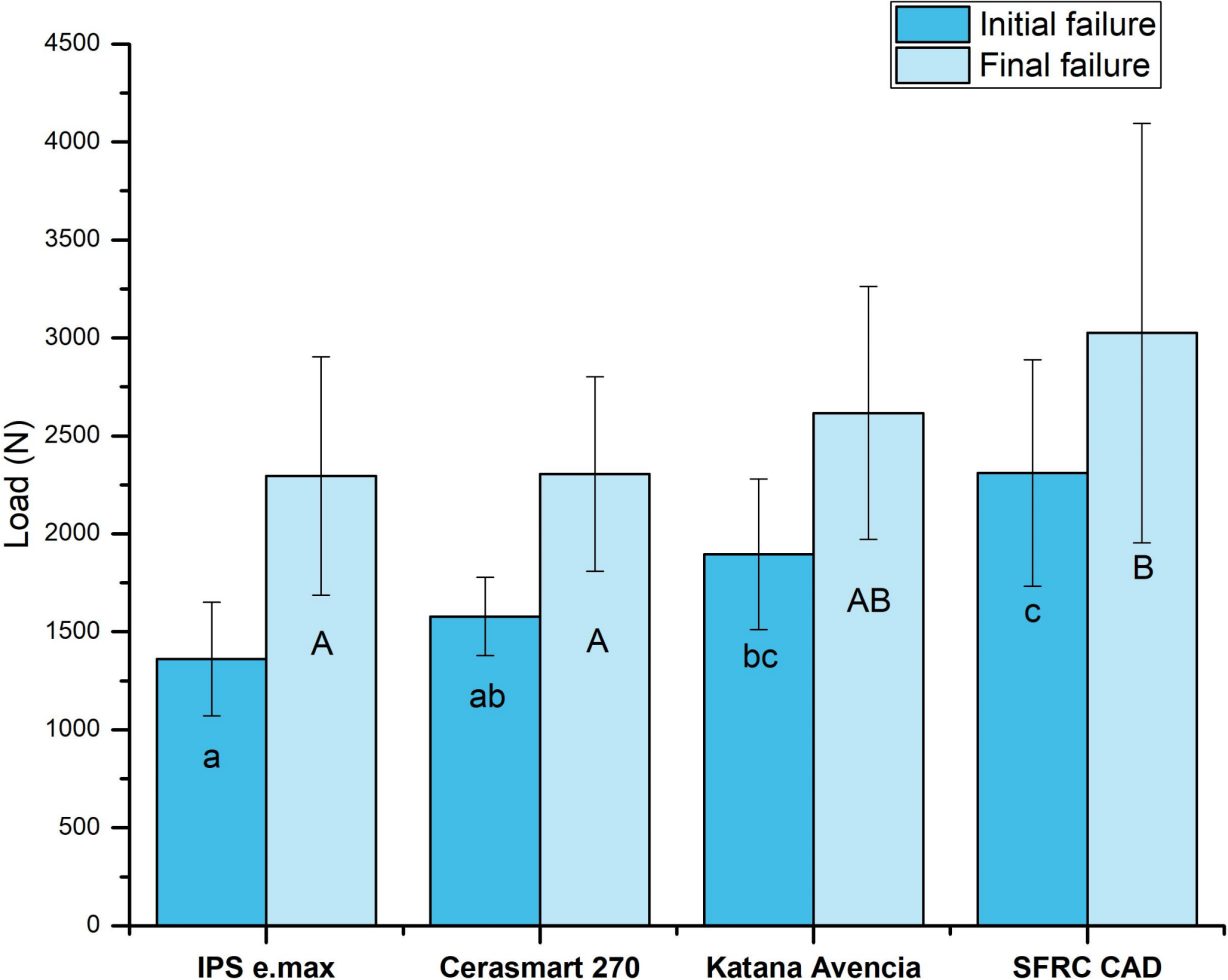
Mean load to initial and final failure (N) and standard deviation (SD) of tested endocrown restorations. The same lowercase letter in the initial failure and uppercase letter in the final failure indicate non-statistically significant differences (*p* > 0.05)

None of the restored teeth failed during the 1.000.000 cycles of fatigue aging. Therefore, all fatigued restorations were subjected to static loading until failure. The type of material had a significant impact on the fracture resistance of the restorations (*p* < 0.05). Levene’s test confirmed that variances were equal and homogeneous across the groups. Figure 3 presents the average fracture load values (initial and final) along with the standard deviations of the tested restorations.

Restorations made of IPS e.max exhibited the lowest initial failure load values (1361 N), while the experimental SFRC CAD restorations demonstrated the highest initial failure load (2310 N) (*p* < 0.05). However, no significant difference (*p* > 0.05) was observed between the SFRC CAD and Katana Avencia groups.

Regarding the final fracture load, the experimental SFRC CAD restorations displayed the highest fracture load values (3025 N) among the tested groups. In contrast, the fracture load for IPS e.max was 2295 N, for Cerasmart was 2305 N, and for Katana Avencia, was 2617 N. Similar to initial failure load results, no significant difference (*p* > 0.05) was found between the SFRC CAD and Katana Avencia groups.

Within each test group, pairwise comparison revealed that there were significant differences between the initial and final failure loads (*p* < 0.05) except for SFRC CAD (*p* > 0.05).

**Fig. 4:**
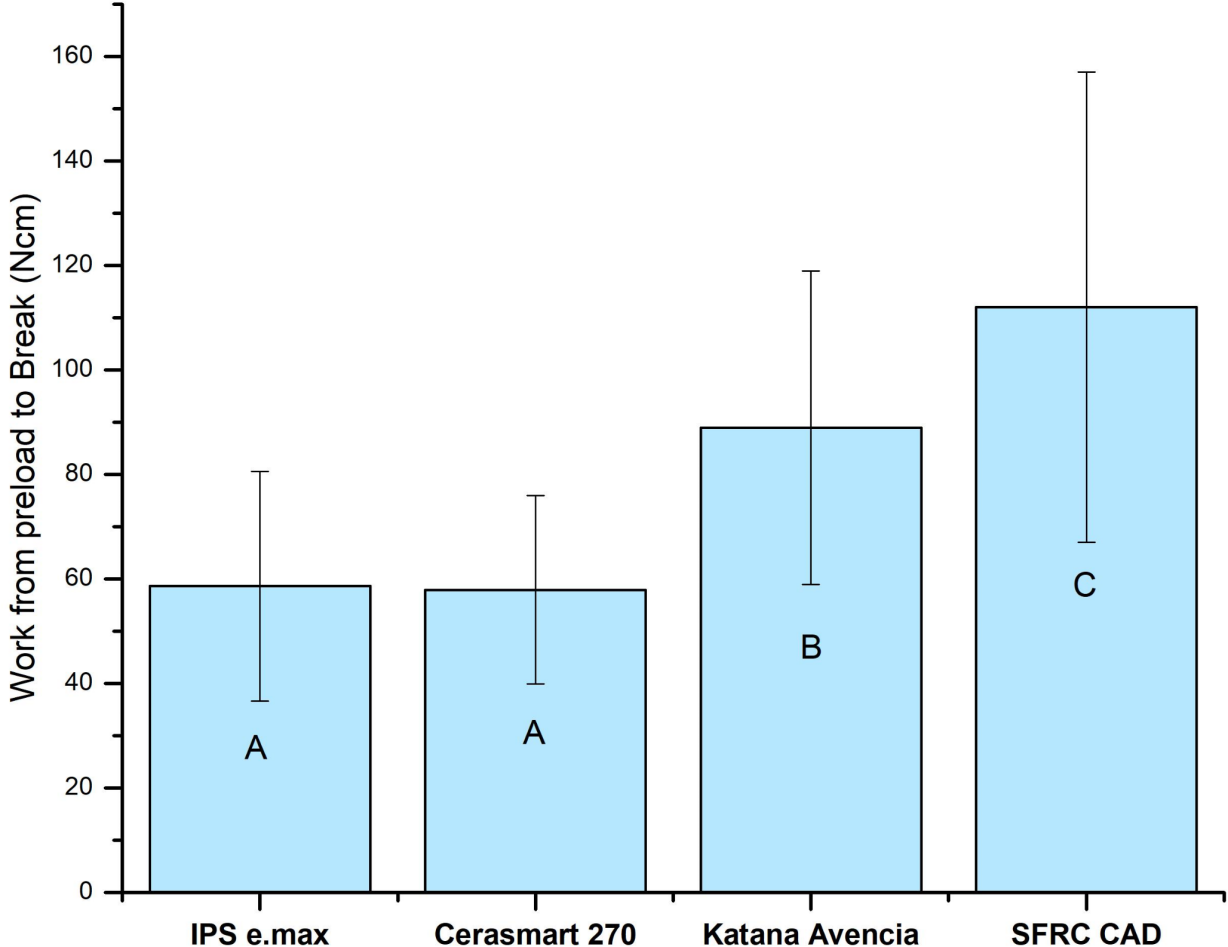
Work of fracture energy (Ncm) from preload to break of tested endocrown restorations. Different letters indicate significant differences (*p* < 0.05)

**Fig. 5:**
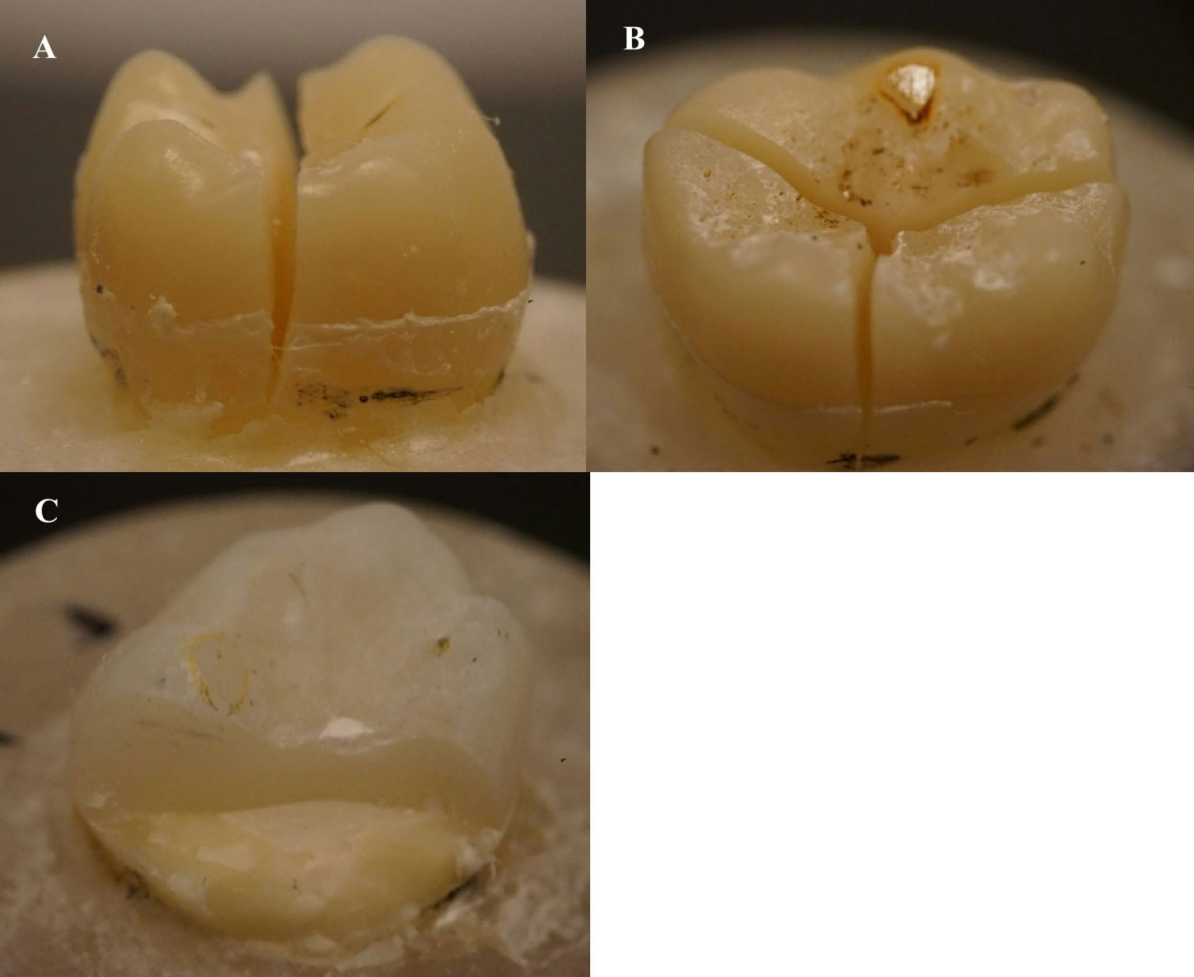
Photographs of fracture modes of tested endocrown restorations. **A** & **B**: Catastrophic vertical fracture of restoration with tooth. **C**: Fracture of only restoration (repairable)

The total energy release (work of fracture) of the restorations was calculated from the area under the load-displacement curves. The contribution of microfibers plays a significant role in increasing the work of fracture energy of SFRC CAD restorations (*p* < 0.05) compared to other tested materials (Figs. 4 and 5).

None of the endocrowns failed adhesively even when significant loading forces were applied. Figure 5 showed the two distinct types of fractures identified through visual inspection of the specimens. As seen in Table 2, fracture of only restoration (repairable) occurred only in the SFRC CAD specimens (47%). Catastrophic fracture extending to root (Fig. 5) occurred predominantly (100%) in conventional CAD/CAM specimens. In this kind of fracture, the tooth is entirely crushed and cannot be restored.

**Table 2:** Distribution of fracture modes among groups

	IPS e.max	Cerasmart 270	Katana Avencia	SFRC CAD
Catastrophic fracture (restoration & tooth)	15	15	15	8
Fracture of only restoration	0	0	0	7

## Discussion

This study examined the fracture resistance of endocrown restorations made from different CAD/CAM materials for teeth with significant structural loss. The null hypothesis that SFRC CAD endocrowns would exhibit the same loading behavior as other CAD/CAM endocrowns was rejected, as material selection had a significant impact on fracture load results.

Interestingly, none of the restored teeth failed during cyclic fatigue aging, suggesting that the restorations could withstand prolonged mechanical stress. The relatively low fatigue load of 150 N, combined with water immersion during testing, likely contributed to their durability. Throughout the aging process, specimens were stored in water at 37 °C for several days. Research suggests that wet conditions can slow fatigue crack development and significantly reduce the risk of failure [26, 27]. Water absorption by the matrix may lead to plasticization, which can blunt fatigue crack tips, reduce stress concentration, and generate residual compressive stress. Additionally, the release of polymerization-induced tensile stress may further slow crack propagation and enhance the material’s load-bearing capacity. However, the effects of plasticization on composite fracture properties remain debated, as some studies suggest that prolonged exposure to an aqueous environment may weaken polymer chains and reduce the composite’s resistance to crack propagation [28].

In this study, both the initial and final failure loads of the endocrown restorations were carefully measured to assess their performance under quasi-static loading. The results showed a clear distinction between initial and final failure loads for all restorations; however, in the SFRC CAD group, the difference was not statistically significant. This could be attributed to the presence of short fibers, which may enhance crack propagation resistance and contribute to a more gradual failure process. The fiber-reinforced structure likely distributes stress more effectively, reducing the abrupt load drop between initial and final failure. Initial failure provides insight into the first signs of weakness, cracking or damage [29]. Therefore, initial failure may be a more sensitive and useful indicator than final failure for in vitro strength evaluation and potential clinical interpretation.

The loading results showed that SFRC CAD endocrowns had the highest fracture load values (initial and final). However, there was no significant difference between the Katana Avencia and SFRC CAD endocrown groups. These findings are consistent with other studies reporting that SFRC CAD has significantly better fracture behavior compared to various ceramic and resin composite materials [24–26]. Several explanations for this finding are available in the literature. Higher fracture load values may result from stress being transferred from the polymer matrix to the glass fibers. The glass fibers individually deflect crack propagation, requiring more energy for cracks to spread throughout the polymer matrix [30, 31]. As a consequence, an increase work of fracture energy can be expected (Fig. 4). In other words, IPS e.max, Cerasmart 270, and Katana Avencia have relatively low fracture toughness and high brittleness compared to SFRC CAD [24, 32, 33]. The ability of a material to withstand damage is determined by its fracture toughness, which measures its resistance to the catastrophic propagation of cracks under applied force [34, 35]. For SFRC CAD blocks, the calculated fracture toughness is 2.9 MPa m^1/2^. In comparison, the fracture toughness of resin composite-based and lithium silicate materials ranges from 1.4 to 2.1 MPa m^1/2^ [24, 32, 33].

El-Damanhoury et al., [36] evaluated the fracture resistance of CAD/CAM endocrown restorations fabricated with feldspathic porcelain (CEREC Blocks), lithium disilicate ceramic (IPS e.max), and hybrid ceramic (Lava Ultimate) and reported that the hybrid ceramic showed significantly higher fracture resistance and a more favorable fracture mode (i.e., fracture of the endocrown without tooth fracture) than CEREC Blocks and IPS e.max. These results are consistent with the results of our study.

Interestingly, Katana Avencia showed higher fracture load values and greater fracture energy compared to Cerasmart 270 (Figs. 3 and 4). This difference may be due to the filler press and monomer infiltration method used to produce Katana Avencia blocks [37], which likely impacts the uniformity and mechanical properties of the blocks [38]. In contrast, Yamaguchi et al., found no significant difference in fracture load values between crowns made from Katana Avencia and Cerasmart CAD/CAM blocks [39]. Given that masticatory forces in the posterior molar region can reach up to 900 N [40], the materials evaluated in this study can withstand fracture loads well above those typical masticatory forces.

The fracture behavior of experimental SFRC CAD restorations was visually analyzed, revealing two types of failures: fractures involving only the restoration and catastrophic failures involving both the tooth structure and the restoration (Table 2). In contrast, the other groups exhibited only catastrophic failures. Fractured specimens showed clear subsurface and surface damage related to loading in the occlusal area. Research suggests that high loading stresses on restorations can cause marginal irregularities, microfractures, or cracks in the luting cement, potentially leading to void formation and catastrophic failure at the interface [41, 42]. However, SFRC CAD restorations have been shown to mitigate this weakness by achieving a reliable bond and minimizing marginal irregularities compared to other commercial CAD/CAM materials [22].

Even though the restorations were subjected to loads greater than the forces of chewing, none of them experienced adhesive failure, which accounts for the strong bond and retention that was formed. The interplay of chemical bonding through primer application and micromechanical interlocking via acid etching explains the cohesiveness between luting cement and CAD/CAM materials. In our study, the parts of the endocrowns extending into the pulp cavity were prepared at a depth of 4 mm. A laboratory studies of 2 and 4 mm depth endocrowns revealed no significant variations in their fracture strength [43] or survival rate [44], however the flat overlays not having a pulpal retentive mean produced an unfavorable result [44].

It is important to highlight that the experimental SFRC CAD composite block has certain limitations. Due to its polymeric nature, it exhibits greater susceptibility to surface deformation and potential discoloration. Additionally, compared to ceramics, the experimental material may demonstrate lower gloss and translucency, which might limit its application in highly esthetic restorations. Another limitation is the lack of thermocycling in the aging process, which could have further simulated the effects of temperature fluctuations in the oral environment. Future laboratory and clinical studies are essential to further evaluate the performance and long-term durability of SFRC CAD material. Comprehensive investigations will help to better understand their performance and clinical outcomes in various restorative applications.

## Conclusion

The type of CAD/CAM restorative material plays a significant role in determining the fracture resistance of endocrown restorations. The findings of this study suggest that SFRC CAD blocks exhibit promising load-bearing capacity, making them a potential alternative to conventional CAD/CAM materials for endocrown applications. However, further in vitro and clinical studies are needed to fully evaluate their long-term performance, durability, and suitability for widespread clinical use.

## Data Availability

No datasets were generated or analysed during the current study.
